# Bench to bedside radiosensitizer development strategy for newly diagnosed glioblastoma

**DOI:** 10.1186/s13014-021-01918-y

**Published:** 2021-09-28

**Authors:** Charlotte Degorre, Philip Tofilon, Kevin Camphausen, Peter Mathen

**Affiliations:** Radiation Oncology Branch, National Cancer Institute, Bldg. 10, Rm B2-3500, 9000 Rockville Pike, Bethesda, MD 20892 USA

**Keywords:** Glioblastoma, Radiosensitizers, Pre-clinical/clinical studies

## Abstract

Glioblastoma is the most common primary brain malignancy and carries with it a poor prognosis. New agents are urgently needed, however nearly all Phase III trials of GBM patients of the past 25 years have failed to demonstrate improvement in outcomes. In 2019, the National Cancer Institute Clinical Trials and Translational Research Advisory Committee (CTAC) Glioblastoma Working Group (GBM WG) identified 5 broad areas of research thought to be important in the development of new herapeutics for GBM. Among those was optimizing radioresponse for GBM in situ*.* One such strategy to increase radiation efficacy is the addition of a radiosensitizer to improve the therapeutic ratio by enhancing tumor sensitivity while ideally having minimal to no effect on normal tissue. Historically the majority of trials using radiosensitizers have been unsuccessful, but they provide important guidance in what is required to develop agents more efficiently. Improved target selection is essential for a drug to provide maximal benefit, and once that target is identified it must be validated through pre-clinical studies. Careful selection of appropriate in vitro and in vivo models to demonstrate increased radiosensitivity and suitable bioavailability are then necessary to prove that a drug warrants advancement to clinical investigation. Once investigational agents are validated pre-clinically, patient trials require consistency both in terms of planning study design as well as reporting efficacy and toxicity in order to assess the potential benefit of the drug. Through this paper we hope to outline strategies for developing effective radiosensitizers against GBM using as models the examples of XPO1 inhibitors and HDAC inhibitors developed from our own lab.

## Background

Glioblastoma (GBM) is the most common primary brain malignancy in adults. Though the Stupp trial established maximal safe resection followed by radiation (RT) with concurrent and adjuvant temozolomide (TMZ) as the standard of care in treating these patients, outcomes remain poor, with median survival of 15 months [1]. In 2019, the National Cancer Institute Clinical Trials and Translational Research Advisory Committee (CTAC) Glioblastoma Working Group (GBM WG) identified 5 broad areas of research thought to be important in the development of new therapeutics for GBM [2]. One of these areas was the improvement in the radioresponse of GBM tumors in situ. Improving local control by enhancing the radioresponse is an important area of research, as the most common area of recurrence is within the high-dose RT field. Attempts at improving RT efficacy using altered fractionation or methods of local boost to increase the radiation dose, have not only failed to demonstrate improvement in survival but have also resulted in an increased toxicity manifested as higher rates of reoperation and radionecrosis [3, 4]. An alternate strategy to improving the response to RT is the addition of radiosensitizers to improve the therapeutic ratio of radiation treatment by increasing tumor sensitivity to radiation without increasing the harm to normal tissues [5–7].

Though the use of radiosensitizers represents a promising strategy, the development of these novel agents has been underwhelming. A review of phase III trials studying systemic agents in GBM found that of seven trials performed on newly diagnosed patients from 1991 to 2016, only the addition of TMZ resulted in a statistically significant improvement in survival [8]. However, because the Stupp trial added TMZ to both the concurrent and adjuvant phases it is unknown if the concurrent TMZ acted as a radiation modifier. There are several proposed explanations for the lack of success of radiosensitizers including the absence of tumor molecular data leading to unknown target availability, the lack of pharmacodynamic testing leading to unknown degree of inhibition, the use of imaging criteria as a surrogate endpoint, and the suboptimal design of the preceding phase II studies [9]. An additional explanation that will be examined in this article is whether the improper selection of drugs tested in phase III trials led to the failures [10]. For many of these trials there was no pre-clinical data, no preceding successful Phase II study, or no Phase II study at all preceding the Phase III studies. Herein, we intend to outline development strategies and the required minimal reporting data needed to progress from bench discovery to a successful Phase III study of a radiation sensitizer in GBM using examples from our own lab.

## Pre-clinical evaluation

Most clinical trials have failed to improve GBM patient outcomes due to the lack of drug efficacy and/or high drug toxicity. To improve the success of GBM clinical trials and avoid exposure of patients to potential toxic therapy, pre-clinical optimization of the treatment is critical. Laboratory studies can help inform the decision of whether a treatment can go from the bench to the bedside by collecting data on the feasibility, safety, and efficacy before beginning the Phase I trial. Target identification and validation are two crucial steps in the drug discovery process. Moreover, the use of in vitro and in vivo models that are the best representation of the patient’s tumor are necessary.

### Target identification

Target identification is the first step in the development of a new drug/therapy since past experiences have indicated that improper target selection is associated with the high failure rate of drug discovery. GBM was one of the first tumor types sequenced by the TGCA allowing a view of its mutational spectrum and copy number variation. Most of the mutated genes code for proteins implicated in the regulation of core signaling pathways that control cell growth and are known to play a role in resistance to treatment, examples of which include PI3K and DNA damage repair pathways or inactivation of p53 and Rb pathways [11]. This catalog of alterations and pathways allow identification of proteins possibly implicated in resistance to radiation and could represent potential therapeutic targets in the development of radiosensitizers.

Another approach to identify potential targets is the use of drug or functional screening using small molecule libraries or RNA/CRISPR interference technologies (RNAi, CRISPRi). For instance, siRNA or shRNA screening on GBM cells has identified PLK1 and PTGFRN as potential targets to enhance GBM radiation sensitivity [12, 13]. Likewise, by using CRISPRi to screen 5689 long non-coding RNA (lncRNA) loci in human GBM cells, a recent study identified lncGRS-1 as a new therapeutic target to enhance GBM radiation response [14]. The compound lumefantrine, a selective inhibitor of the protein Fli-1, was identified using a small molecule library [15]. Once identified, a target needs to be validated to elucidate its role in enhancing the radiation induced killing of tumor cells and its potential off-target effects on normal cells.

### Target validation

In vitro studies are the starting point in drug discovery and validation as well as the preliminary evaluation of drug/radiation combination. These models typically include the use of GBM cell lines, such as U87 or U251, where techniques such as gene expression modulation or screening are straightforward. However, these traditional cell lines, grown in serum supplemented media, have been shown to have little in common with the biology of GBM in situ [16]. Therefore, although they are easy to manipulate and widely used, data generated using these traditional GBM cell lines in drug discovery should be interpreted carefully. About 10 years ago, GBMs were shown to include a minor subpopulation of clonogenic cells referred to as Glioma Stem-like Cells (GSCs). These cells, isolated from glioblastoma surgical specimens, have several in vitro properties in common with normal neural stem cells including continuous self-renewal, expression of stem cell markers, and at least partial differentiation along neuronal and glial pathways. Moreover, brain tumor xenografts initiated in immunocompromised mice from GSCs have been shown to replicate the genotype, phenotype, and in vivo growth patterns of the GBM from which they originated [17]. At present, GSCs are considered a more accurate in vitro model of GBM biology. With respect to the development of radiosensitizers, GSCs can be used in clonogenic assays to determine intrinsic radiation sensitivity. This assay allows the measurement of the capacity of a cell to divide and form a colony after radiation treatment with or without the presence of drugs or genetic/molecular manipulations. The use of in vitro models enables the comparison of GBM cells to normal cells and provides an initial assessment of (1) whether the targeted agent selectively enhances the radiosensitivity of GBM cells over normal cells and (2) its potential for normal cell toxicity.

In vitro models, however, do not fully reproduce the phenotype or genotype of either the patient’s normal tissue nor their tumor and cannot recapitulate the cellular complexity of the tumor microenvironment, which is known to influence radiation sensitivity [17, 18]. Therefore, the use of in vivo models is necessary to complement in vitro pre-clinical studies. GBM cell transplantation in mice is one in vivo model used to investigate tumor radiation response. Though allograft models may be more suitable for tumor immunity and immunotherapeutic research, mouse tumor cells have shown significant differences in terms of fundamental processes regulating radioresponse such as the DNA damage response [19]. As such, orthotopic xenograft models are thought to be more representative of the tumor and its response to therapeutics, especially irradiation. This model allows precise control of spatial and temporal tumor initiation. Furthermore, orthotopic implantation of GSCs instead of GBM cells lines recapitulate some tumor features found in situ such as tumor heterogeneity and the high infiltration into the brain stroma seen in primary tumors. Also, because the tumor is implanted in the brain, it is possible to investigate normal tissue toxicity after radiation and the ability of the drug to cross the blood–brain-barrier (BBB). Nevertheless, despite the use of human GSCs and the correct organ context, the orthotopic xenograft model presents some significant disadvantages such as the lack of an intact immune system and the presence of mouse rather that human stromal cells. Therefore, even the most promising pre-clinical data must be confirmed by demonstrating safety and efficacy through clinical studies in humans.

### Targeting the nuclear export receptor XPO1

The following section delineates an example from our own lab of using pre-clinical evaluation to select and test a potential target for radiosensitization. To generate insight into the processes mediating the radioresponse of GBMs and to provide a potential source of molecular targets for radiosensitization, polysome gene expression profiling was used to define the radiation-induced translatomes for a panel of human GSC lines [20, 21]. Analysis of the radiation-induced translatomes identified a network that regulates nucleus to cytoplasm transport. A major hub in this network was XPO1, a critical nuclear transport receptor mediating the export of a subset of RNAs as well as over 200 proteins [22, 23]. To test the functional significance of XPO1 and nuclear/cytoplasmic transport in radiosensitivity, GSCs were treated with selinexor, a small molecule inhibitor of XPO1.

The radiosensitivity of GSCs was enhanced by selinexor, with a dose enhancement factor (DEF) at 10% survival of 1.46 in NSC11, 1.39 in 0923 and 1.63 in U251 cells. This effect was attributed to an inhibition of the repair of radiation-induced DNA double strand breaks (DSB). Twenty-four hours after irradiation gamma-H2AX foci, a marker of DNA-DSBs, were doubled in NSC11 cells when RT was combined with selinexor and tripled in 0923 cells for the same combination. Whereas initial studies of the anti-cancer actions of selinexor had been generally attributed to a reduction in the nucleus to cytoplasm transport of proteins mediating apoptosis [24], no changes in apoptosis were detected in GSCs after exposure to selinexor and/or radiation, which suggested an alternative mechanism for radiosensitization of GSCs.

In addition to the nuclear export of specific proteins, XPO1 regulates export of ribosomal RNA (rRNA); its inhibition would likely affect the general process of gene translation. Accordingly, selinexor was shown to inhibit rRNA nuclear export in the GSC lines and to reduce translational efficiency from 0.64 to 0.26 in NSC11 cells and from 0.46 to 0.32 in 0923 cells. This suggest that inhibition of translation plays a role in selinexor-induced radiosensitization, which is consistent with previous reports showing that inhibiting translation via reduction in mTOR activity or eIF4E levels inhibited DSB repair and enhanced tumor cell radiosensitivity [25, 26]. Extension of these results to orthotopic xenografts initiated from GSCs revealed that selinexor decreased tumor translational efficiency from 0.55 to 0.43 and significantly enhanced the radiation-induced prolongation of median survival from 9 days for an individual treatment to 18 days for combination treatment. Therefore, in both in vitro and in vivo systems radiosensitization was associated with a decrease in general translational efficiency and results suggest that selinexor delivered in combination with radiotherapy may improve the effectiveness of GBM treatment.

Pre-clinical in vitro and in vivo data about the selinexor look promising in the context of radiosensitizer development for GBM. This drug is the only XPO1 inhibitor already approved by the FDA as a treatment for patients with multiple myeloma and has been tested in multiple clinical trials across numerous malignancies. In regard to GBM treatment, all the pre-clinical results are in favor of a good bioavailability, an enhancement of the radiation sensitivity and low toxicity. Therefore, the next step would be a Phase I clinical trial. Two Phase I clinical trials have begun recently and selinexor will be used for newly diagnosed and recurrent GBM in combination with the standard current treatment comprising TMZ and radiation.

While selinexor is undergoing Phase I investigation, it is important that it demonstrates solid results in order to proceed on to a Phase II study. As with all Phase I studies the primary goal is to assess the safety and maximum tolerated dose of the new agent. Within the protocol’s own study stopping rules, accrual for the trial will be halted if ≥ 2 patients miss consecutive doses of RT or 5 days of temozolomide due to toxicity possibly due to selinexor. However, in progressing to Phase II trial a reasonable level of efficacy of treatment must also be present. While the small sample size of a Phase I trials limit useful interpretation of progression-free and overall survival, a trial agent must at least demonstrate that it’s not significantly worse than the current standard of care before proceeding to more thorough investigations of efficacy.

## Phase II evaluation

As stated above, high-quality Phase II data is essential in picking agents appropriate for Phase III trials. A recent publication by our group reviewed all Phase II trials of potential radiosensitizers in the TMZ-era, examples of which include EGFR inhibitors, histone deacetylase (HDAC) inhibitors and antiangiogenic therapies. Across these papers there were issues with trial design as well as an overall inconsistency of reporting outcomes [27]. Though OS (Overall Survival) was consistently reported, reporting of PFS (Progression Free Survival) and toxicity varied drastically between trials. Of the 14 total trials, all reported OS, 11 trials reported median PFS while 8 reported 6-month PFS. 10 trials reported acute grade 3 and 4 toxicity in absolute numbers, 1 reported the number of > grade3 toxicity, 1 reported the percent of patients who experienced grade 3/4 toxicity and one trial reported toxicity as the percent of patients suffering from grade 3 or 4 toxicity separated into hematological and non-hematological categories. Only one trial reported late toxicity outcomes. Additionally, it is rare for Phase II trials to include a control arm to properly gauge a potential survival benefit of experimental arms. However, there is a trend to run randomized Phase II studies as they require far fewer patients than a full Phase III trial, saving resources and exposing fewer patients to non-beneficial combinations [8]. The downside of a randomized Phase II is that it might not qualify as a registration trial and a Phase III might still have to be conducted.

### Valproic acid as a radiosensitizer

An example from our own group for reporting outcomes of a Phase II study is the addition of Valproic acid (VPA) to RT and concurrent TMZ for the purpose of radiosensitization. VPA inhibits HDAC enzymes that remove acetyl groups from nuclear histones. Pre-clinical data including in vitro clonogenic survival across multiple cell lines, evidence of DNA-DSB repair inhibition, efficacy in an intracranial (IC) model, use of white blood cell (WBC) hyper-acetylation as a biomarker and the known penetration of VPA through the BBB supported the clinical investigation of this drug.

A small Phase II trial tested the addition of VPA to standard RT and TMZ in 37 patients and found that it was a well-tolerated regimen, with only 6 patients experiencing a grade 4 toxicity while on treatment (all blood/bone marrow in nature) [28]. As use of VPA is not uncommon in treatment of GBM for management of seizures, and as the maximum tolerated dose had been established both for epilepsy as well as for treatment of solid tumors, it was decided it was safe to proceed with Phase II investigation. This was the only Phase II trial with late toxicity reported and found only 2 late toxicities greater than grade 2 (one blood/ bone marrow and one pain, both grade 3 in severity). While the number of acute toxicities was seemingly higher compared to the Phase III Stupp data, when compared to the equivalent Phase II data evaluating the addition of TMZ to RT there were actually fewer grade 3/4 toxicities in the study of VPA. Even more remarkably, patients treated with VPA had a Median OS of 29.6 months and 6-months PFS of 70% which represented an improvement over historical controls. MGMT status was evaluated retrospectively in all available patients, and of 15 available patients, 8 tumors were methylated and 7 tumors unmethylated. In keeping with reported literature methylated tumors had improved 1-year OS and PFS (100% vs. 85% and 78% vs. 30% respectively). Additionally, analysis of long-term survivors found that 6 patients lived greater than 3 years following completion of treatment, and in this cohort median OS was 73.8 m and median PFS was 53.1 m [29]. In this cohort of patients, 4 had molecular analysis, 3 patients had MGMT methylated tumors, and one was IDH mutated.

However, despite these promising outcomes, proceeding to a Phase III trial has proved difficult due to a number of barriers. VPA is currently off patent, which can make support for using it for a novel indication difficult. As well, some question if the results of the Phase II are accurate. A large pooled-analysis in patients from the AVAGlio (Avastin and Glioblastoma), CENTRIC (Cilengitide, Temozolomide and Radiation Therapy in treating Radiation Therapy and Methylated Gene Promoter Status), CORE (Cilengitide, Temozolomide and Radiation Therapy in treating Radiation Therapy and Unmethylated Gene Promoter Status) and Radiation Therapy Oncology Group 0825 trials found the use of VPA at anti-epilepsy dosages found no improvement in either OS or PFS. However, the pooled analysis lacked information on the VPA dose. While the Phase II trial had a set protocol for dose and administration, the pooled analysis recorded whether any VPA was administered at baseline, start of chemoradiation or completion of chemoradiation. As such, patients in the pooled analysis were much more likely to receive typical anti-seizure prophylaxis doses of 5–10 mg/kg, than the escalated 25 mg/kg doses required for radiosensitization on the Phase II trial. Additionally, because of the retrospective nature of the pooled analysis, it is impossible to rule out potential biases in the selection of VPA in this population as opposed to other anti-epileptics. Given the promising results and minimal toxicity of the Phase II trial, as well as the failures of other agents with less substantial pre-clinical data, further research is warranted into the use of VPA as a radiosensitizer in GBM.

## Conclusion

As stated by the GBM WG, GBM remains an essentially incurable disease [2]. While the addition of TMZ to standard RT represented a significant step forward for adjuvant treatment, progress since has been lacking and prognosis remains poor. Improving radioresponse through radiosensitizers is a promising approach due to the high rate of local failure. However, in order to develop effective radiosensitizers in an efficient manner, proper development of agents and study design is required. Target identification, strong pre-clinical and clinical data and consistent reporting of end points are all required in order to discern agents that potentially improve outcomes. The results of more recent Phase II trials based on solid pre-clinical data suggests that radiosensitizors remain a promising avenue into improving GBM outcomes.

## Data Availability

All data supporting the results of this review are published in the cited references.

## Abbreviations

AVAGlio: Avastin and Glioblastoma
BBB: Blood–Brain-Barrier
CENTRIC: Cilengitide, Temozolomide and Radiation Therapy in treating Radiation Therapy and Methylated Gene Promoter Status
CORE: Cilengitide, Temozolomide and Radiation Therapy in treating Radiation Therapy and Unmethylated Gene Promoter Status
CTAC: Clinical Trials and Translational Research Advisory Committee,
GBM WG: Glioblastoma Working Group
GBM: Glioblastoma,
GSCs: Glioma Stem-like Cells
HDACs: Histone deacetylases
IC: Intracranial
lncRNA: Long non-coding RNA
MGMT: O6-methylguanine-DNA methyl-transferase
OS: Overall Survival,
PFS: Progression Free Survival
Radiation: RT
rRNA: Ribosomal RNA
TMZ: Temozolomide
VPA: Valproic acid
WBC: White blood cell
